# Demystifying Grief in the Dementia Divide: A Case for Grief Therapy in Dementia Care

**DOI:** 10.1093/geroni/igaa057.3241

**Published:** 2020-12-16

**Authors:** Adrienne Ione

**Affiliations:** Silver Linings Integrative Health, LLC, Tacoma, Washington, United States

## Abstract

The results of emotional and psychological losses overlap with behavioral and psychological symptoms of dementia (BPSD) in the neuronal and structural changes in the brain. The aim of this current study was to explore the magnifying effects of COVID-19 on exacerbating residual losses, illuminating a case for using grief therapy to moderate BPSD. A case control study was conducted with people ages 65 and greater, with an established diagnosis of dementia prior to March 2020. Compared with an active control group - participants without a current dementia diagnosis who self-reported mild cognitive shifts and who also received active grief-informed therapies – offer supporting evidence of a strong factor of efficacy for including grief therapy in services offered to people living with dementia. Evidence of a continued point improvement on both the brief grief questionnaire and inventory of complicated grief, as well as decreased severity of items on NPI-Q corroborate this therapeutic recommendation. Now more than ever – as people across the globe who are diagnosed with dementia face uncertain ramifications of previous grief episodes, ones that have potentially been reignited by the flames of COVID-19 – therapists must foster safe spaces informed by novel therapeutic grief approaches. In any just society, emphasis on therapeutic techniques that allow participants to ventilate their feelings and fears, as well as promote movement along a continuum from isolation to intimacy, must prevail. People exhibiting BPSD should not be excluded from such treatments.

